# Antimicrobial Resistance Patterns of *Enterobacter cloacae* and *Klebsiella aerogenes* Strains Isolated from Clinical Specimens: A Twenty-Year Surveillance Study

**DOI:** 10.3390/antibiotics12040775

**Published:** 2023-04-18

**Authors:** Jari Intra, Davide Carcione, Roberta Maria Sala, Claudia Siracusa, Paolo Brambilla, Valerio Leoni

**Affiliations:** 1 Clinical Chemistry Laboratory, Fondazione IRCCS San Gerardo Dei Tintori, via Pergolesi 33, 20900 Monza, MB, Italy; 2 Laboratory of Medicine and Microbiology, Busto Arsizio Hospital—ASST Valle Olona, via Arnaldo da Brescia, 1, 21052 Busto Arsizio, VA, Italy; 3 Department of Laboratory Medicine, University of Milano-Bicocca, Azienda Socio Sanitaria Territoriale della Brianza, ASST-Brianza, Desio Hospital, via Mazzini 1, 20833 Desio, MB, Italy

**Keywords:** antibiotic resistance, *Enterobacter cloacae*, *Klebsiella aerogenes*, epidemiology, fosfomycin, surveillance study

## Abstract

We retrospectively analyzed the antimicrobial data of *Enterobacter* spp. strains isolated from hospitalized subjects and outpatients over 20 years (2000–2019). A total of 2277 non-duplicate *Enterobacter* spp. isolates, 1037 from outpatients (45%) and 1240 from hospitalized subjects (55%), were retrieved. Most of samples are infections of the urinary tract. Considering *Enterobacter aerogenes,* now classified as *Klebsiella aerogenes,* and *Enterobacter cloacae*, representing more than 90% of all isolates, except for aminoglycosides and fluroquinolones, which showed significant antibiotic decreasing trends (*p* < 0.01), none of the other antimicrobial agents tested showed significant changes in both groups (*p* > 0.05). Conversely, there was a significant increasing resistance trend for fosfomycin (*p* < 0.01), among both community and hospital-related subjects, most probably owing to uncontrolled and improper usage. Surveillance studies on antibiotic resistance at the local and regional level are required to detect new resistance mechanisms, reduce inappropriate antimicrobial consumption, and increase the focus on antimicrobial stewardship.

## 1. Introduction

The improper and unnecessary use of antibiotics and the limited availability of new antimicrobial agents have contributed to the rise in antimicrobial resistance, which represents one of the most dangerous public human health problems in the world [1,2,3,4]. Surveillance studies allow the monitoring of the emergence of antibiotic resistances, the introduction of control measures, and the guidance for antimicrobial use in patients. *Enterobacter* species are Gram-negative rod-shaped opportunistic microorganisms belonging to the family *Enterobacteriaceae*, and cause severe urinary tract infections (UTIs), septicemia, and pneumonia in subjects who are on mechanical ventilation in the intensive care unit (ICU), premature infants, patients with diabetes, traumatized patients, patients affected by leukemia, or those under immunosuppressive therapy [5,6,7]. *Enterobacter* species are members of the ESKAPE group (*Enterococcus faecium*, *Staphylococcus aureus*, *Klebsiella pneumoniae*, *Acinetobacter baumannii*, *Pseudomonas aeruginosa*, and *Enterobacter* species), which are known to be the most significant causes of nosocomial infections. Therefore, the World Health Organization (WHO) placed *Enterobacter* spp. onto the priority list of microorganisms for which it is essential to research and develop new antibiotics [6]. Actually, out of 22 species belonging to the *Enterobacter* genus, such as *E. aerogenes*, *E. amnigenus*, *E. arachidis*, *E. asburiae*, *E. carcinogenus*, *E. cloacae*, *E. cowanii*, *E. dissolvans*, *E. gergoviae*, *E. helveticus*, *E. hormaechei*, *E. kobei*, *E. ludwigii*, *E. mori*, *E. nimipressuralis*, *E. oryzae*, *E. pulveris*, *E. pyrinus*, *E. radicincitans*, *E. soli*, *E. taylorae*, and *E. turicensis*, two of them, *Enterobacter aerogenes*, recently classified as *Klebsiella aerogenes* after full-genome sequence analysis, and *Enterobacter cloacae*, are responsible of the majority of human diseases [6]. *Enterobacter* spp. present an intrinsic resistance to ampicillin and broad-spectrum cephalosporins and, through the acquisition of genetic mobile elements, have become resistant to many antibiotics, such as third-generation cephalosporins and carbapenems, thus making difficult the selection of an appropriate therapy [5,6,7,8,9]. The increasing trends of antibiotic resistance of *Enterobacter* strains are associated with higher mortality rates in infected subjects, longer hospitalizations, and higher costs of treatments [6]. A few works on antibiotic resistances of *Enterobacter* spp. have been published, particularly over a long time period [10,11,12,13]. In this study, we aimed to retrospectively investigate the susceptibility patterns of *Klebsiella aerogenes* (*Enterobacter aerogenes*) and *Enterobacter cloacae* strains using the recorded microbiological data of the Laboratory of Italian Hospital of Desio over a 20-year period (2000–2019). The assessment of antimicrobial resistance trends might be helpful to clinicians to prescribe a more appropriate therapy and to reduce treatment failure in patients.

## 2. Results

**Figure 1:**
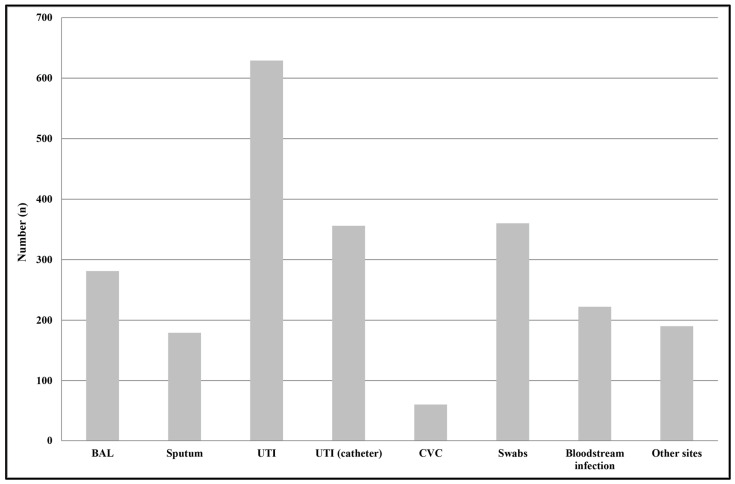
Distribution of *Enterobacter* spp. isolates by infection type. Swabs were from different anatomical sources, such as the nose, throat, groin, hears, and wounds. Other sites included samples from ascetic and peritoneal fluids. Abbreviations: BAL, Bronchoalveolar lavage; UTI, urinary tract infections; UTI (catheter), catheter-associated urinary tract infection; CVC, central venous catheter.

We identified a total of 2277 non-duplicate *Enterobacter* spp. strains from positive samples, 1037 from outpatients (45%), and 1240 from hospitalized subjects (55%). Among inpatients, most of *Enterobacter* spp. strains were isolated from surgery (*n* = 207), followed by ICU (*n* = 162), neurology (*n* = 162), and internal medicine (*n* = 156). The median age of patients was 69 years (interquartile range (IQR): 4–87 years). The majority of isolates were from females (83% compared with 17% males). The most common specimen type from which *Enterobacter* spp. strains were isolated was midstream urines (28%, *n* = 629), followed by swabs from different sources, particularly the skin, vagina, and urethra (16%, *n* = 360); urine samples from subjects with a catheter (16%, *n* = 356); bronchoalveolar lavage (BAL) (12%, *n* = 281); bloodstream (10%, *n* = 222); and sputum (8%, *n* = 179) (Figure 1).

**Figure 2:**
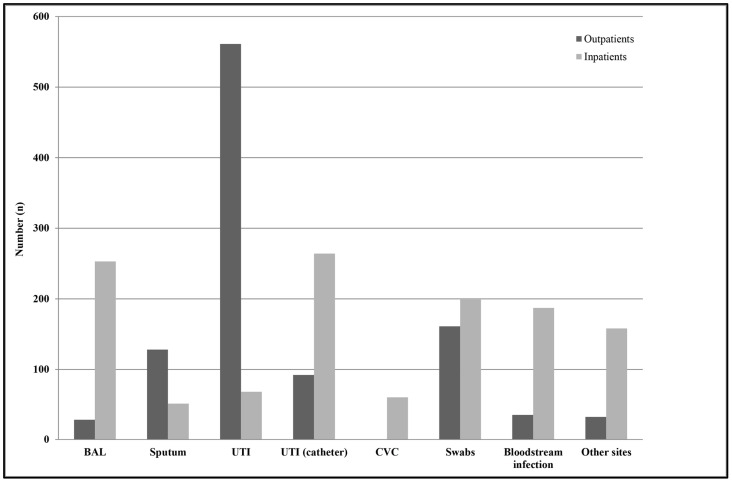
Distribution of *Enterobacter* spp. isolates by infection type among outpatients and hospitalized subjects. Swabs were from different anatomical sources, such as the nose, throat, groin, hears, and wounds. Other sites included samples from ascetic and peritoneal fluids. Abbreviations: BAL, Bronchoalveolar lavage; UTI, urinary tract infections; UTI (catheter), catheter-associated urinary tract infection; CVC, central venous catheter.

Among outpatients, the most common specimen type from which *Enterobacter* spp. strains were isolated was midstream urine (54%, *n* = 561), while, among inpatients, the most common specimen types were urine samples from subjects with a catheter (21%, *n* = 264) and BAL (20%, *n* = 253) (Figure 2). Among all of the isolates, *E. cloacae* was identified in 1230 samples; *K. aerogenes* (*E. aerogenes*) in 907; and *E. amnigenus*, *E. kobei*, *E. asburiae*, and *E. gergoviae* in 29; while in 111 specimens, it was not possible to determine the species using either VITEK^®^ 1 and 2 systems or Vitek^®^ MALDI-TOF MS (bioMérieux, Marcy l’Étoile, France), thus they were reported as *Enterobacter* spp. No significant differences were observed between inpatients and outpatients concerning the species isolated (*p* > 0.05). A significant decrease was observed in the isolation of *E. cloacae* strains, from a mean value of 60 cases/year to 20 cases/year in the last two years (2018 and 2019) (*p* < 0.01), while the number of *K. aerogenes* (*E. aerogenes*) isolates remained constant over the whole period (45 cases/year).

**Figure 3:**
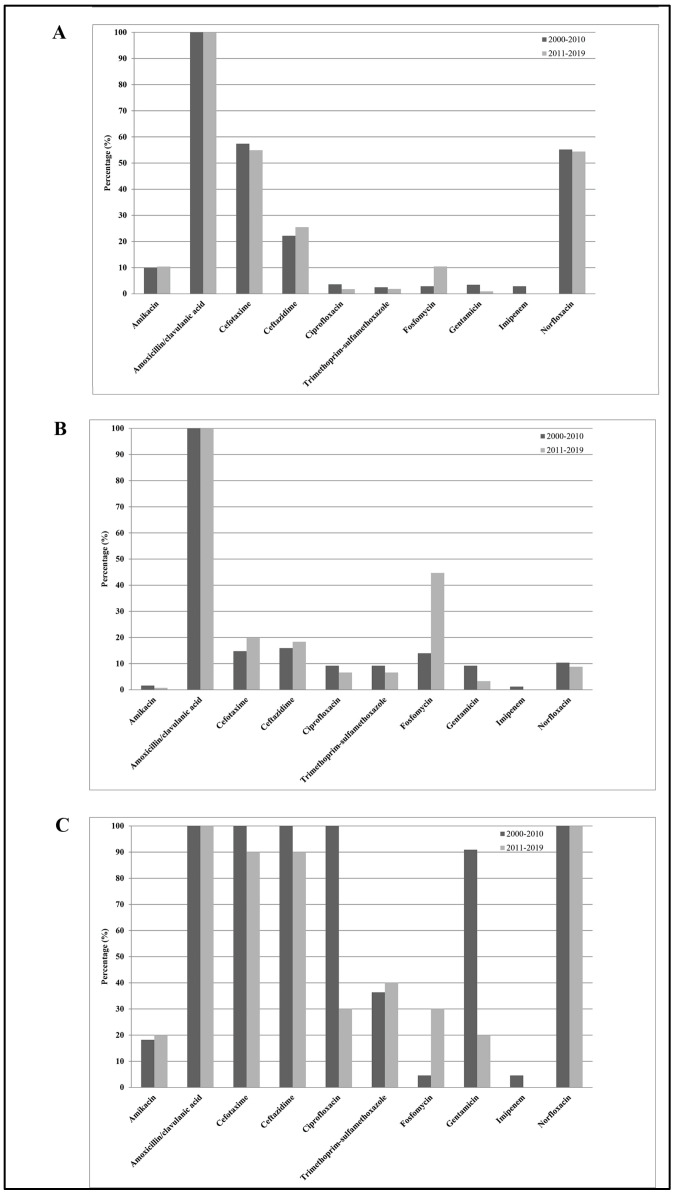
Distribution of mean antibiotic resistance percentages of *Enterobacter cloacae* isolates against the 10 antimicrobial agents tested, amikacin, amoxicillin/clavulanic acid, cefotaxime, ceftazidime, ciprofloxacin, norfloxacin, fosfomycin, gentamicin, imipenem, and trimethoprim-sulfamethoxazole, among all subjects enrolled in the study (**A**), among outpatients (**B**), and among hospitalized subjects (**C**) during the study periods 2000–2010 and 2011–2019.

Figure 3 shows the *E. cloacae* susceptibilities to the 10 antimicrobial agents tested among all subjects enrolled in the study. From 2000–2010 to 2011–2019, except for gentamicin (from 50% to 12%) and ciprofloxacin (from 55% to 18%), which showed significant decreasing resistance rates (*p* trend < 0.01), and except for fosfomycin (from 9% to 37%), which showed a significant increasing resistance rate (*p* trend < 0.01), none of the other antibiotics (mean values: amikacin 10%, amoxicillin/clavulanic acid 100%, cefotaxime 56%, ceftazidime 56%, imipenem 1%, and trimethoprim-sulfamethoxazole 23%) showed significant changes over time (*p* trend > 0.05). Considering all antibiotics, the mean resistance rate increased from 42% in 2000–2010 to 36% in 2011–2019, representing a not statistically significant difference (*p* trend > 0.05). However, separating the entire population into inpatients and outpatients, we noted the following: (1) *E. cloacae* strains isolated from inpatients were resistant to two or more categories of antibiotics (*β-lactams*, third-generation cephalosporins, and *fluoroquinolones*) compared with those isolated from outpatients; (2) significantly decreased resistance rate values for ciprofloxacin and gentamicin from 2000–2010 to 2011–2019 in hospitalized subjects (*p* trend < 0.01); (3) significantly increased levels of fosfomycin in the two groups (*p* trend < 0.01); and (4) not significant changes over time for the other antibiotics tested (*p* trend > 0.05).

**Figure 4:**
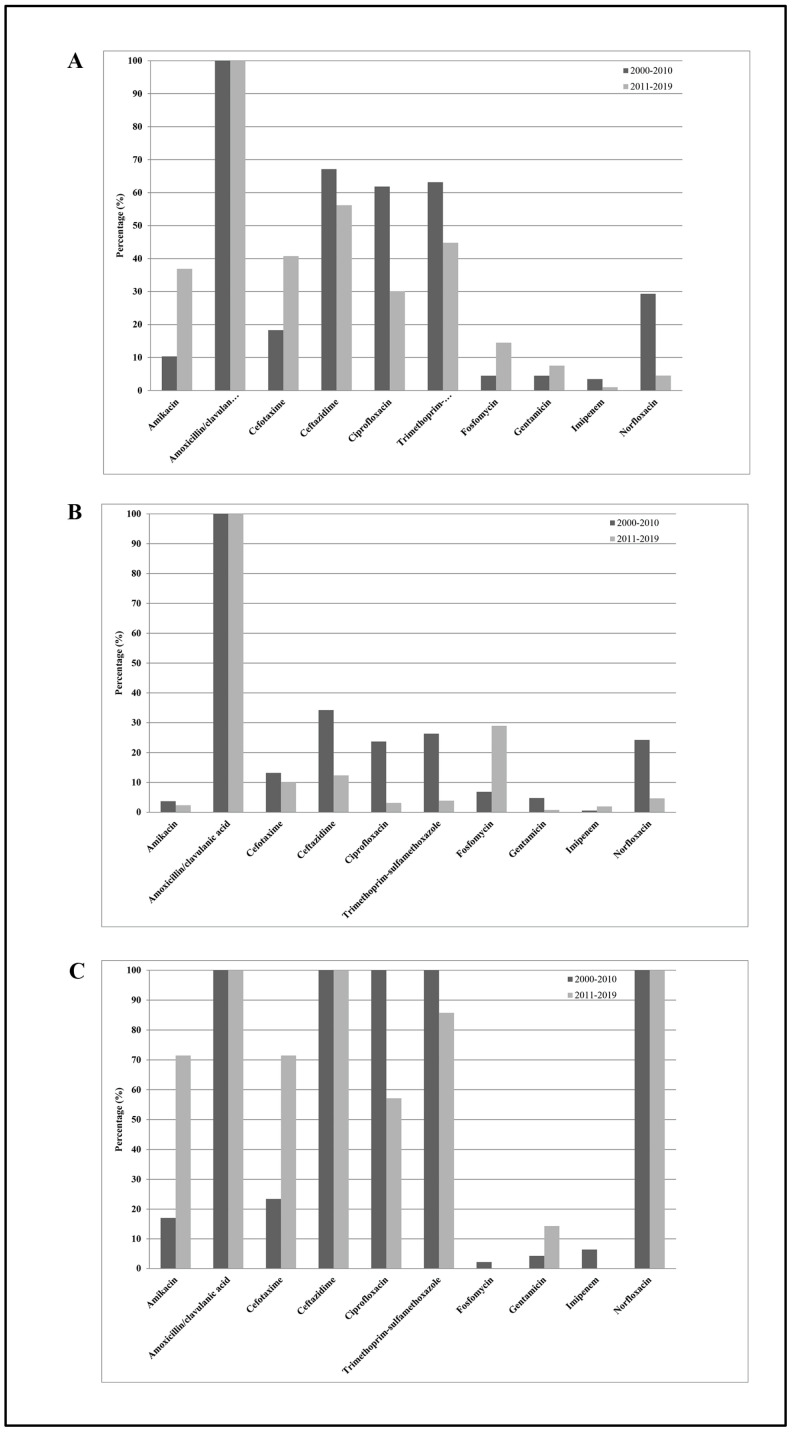
Distribution of mean antibiotic resistance percentages of *K. aerogenes* (*E. aerogenes*) isolates against the 10 antimicrobial agents tested, amikacin, amoxicillin/clavulanic acid, cefotaxime, ceftazidime, ciprofloxacin, norfloxacin, fosfomycin, gentamicin, imipenem, and trimethoprim-sulfamethoxazole, among all subjects enrolled in the study (**A**), among outpatients (**B**), and among hospitalized subjects (**C**) during the study periods 2000–2010 and 2011–2019.

Figure 4 shows the *K. aerogenes* (*E. aerogenes*) susceptibilities to the 10 antimicrobial agents tested among all subjects enrolled in the study. From 2000–2010 to 2011–2019, except for amikacin (from 10% to 37%), trimethoprim-sulfamethoxazole (from 63% to 45%), ciprofloxacin (from 62% to 30%), norfloxacin (from 62% to 52%), and ceftazidime (from 67% to 56%), which showed significantly decreasing resistance rates (*p* trend < 0.01), and except for cefotaxime (from 18% to 41%) and fosfomycin (from 4% to 14%), which showed a significantly increasing resistance rate (*p* trend < 0.01), none of the other antibiotics (mean values: amoxicillin/clavulanic acid 100%, gentamicin 6%, and imipenem 1%) showed significant changes over time (*p* trend > 0.05). Considering all antibiotics, the mean resistance rate increased from 36% in 2000–2010 to 34% in 2011–2019, representing a not statistically significant difference (*p* trend > 0.05). On the other hand, among outpatients, the mean resistance rate decreased from 24% in 2000–2010 to 17% in 2011–2019, representing a statistically significant difference (*p* trend < 0.01). Separating the entire population into inpatients and outpatients, we observed the following: (1) *K. aerogenes* (*E. aerogenes*) strains isolated from inpatients were resistant to two or more categories of antibiotics (*β-lactams*, third-generation cephalosporins, *fluoroquinolones*, and aminoglycosides) compared with those isolated from outpatients; (2) significantly decreased resistance rate values for ciprofloxacin in hospitalized subjects (*p* trend < 0.01); (3) significantly decreased resistance rate values for *fluoroquinolones,* ceftazidime, and trimethoprim-sulfamethoxazole in outpatients (*p* trend < 0.01); (4) significantly increased levels of fosfomycin in outpatients (*p* trend < 0.01); (5) significantly increased levels of *aminoglycosides* and cefotaxime in inpatients (*p* trend < 0.01); and (6) not significant changes over time for the other antibiotics tested (*p* trend > 0.05).

Among all 2277 *Enterobacter* spp. strains, we observed a significant increase in *E. cloacae*-producing ESBL isolates and a significant decrease in *K. aerogenes* (*E. aerogenes*)-producing ESBLs among both inpatients and outpatients over the study period (*p* trend < 0.01), as well as a constant low value of carbapenem-resistant *Enterobacter* spp. isolates in the period from 2000–2010 to 2011–2019 (0.5%).

## 3. Discussion

*Enterobacter* species are present in the normal flora of the human gastrointestinal tract and are also widely encountered in the environment. These bacteria are found in the soil, sewage, and water and are considered phytopathogens for several plant species. Moreover, some species are used in bioprocessing and metabolic engineering approaches. Few data are available about the pathogenicity and virulence factors of *Enterobacter* spp. owing to the low number of studies. It is known that *Enterobacter* spp. possess a flagellum and they have the ability to form biofilm. They produce different endotoxins, enterotoxins, alpha-hemolysins, and cytotoxins [6]. Recently, they have emerged as important agents of respiratory tract infections, particularly in subjects on mechanical ventilation, as well as of UTI, mainly in subjects with a catheter [6,9]. About 5% of the bacteremia acquired in the hospital, 5% of pneumonia, 4% of UTIs, and 10% of peritonitis are caused by *Enterobacter* spp. [6]. We assessed the antimicrobial resistance trends among *Enterobacter* strains from both inpatients and outpatients between 2000 and 2019. In our study, *E. cloacae* and *K. aerogenes* (*E. aerogenes*) strains accounted for 94% of all isolates, similar to the findings reported in previous work [6,9]. Out of all isolates, most of them were obtained from urine samples either from midstream or catheter-associated urine specimens, as previously described [6,9]. The worldwide overuse, the inappropriate prescription of antibiotics, and the lack of development of new antibiotic agents by pharmaceutical companies have promoted the rapid emergence of antimicrobial resistance in bacteria, causing the death of nearly 700,000 people worldwide every year [14]. Surveillance studies are generally seen as primary strategies in the identification of bacterial changes in antimicrobial susceptibilities, with the aim to critically review the empirical treatment protocols. The present work is one of the largest databases on susceptibility patterns of *Enterobacter* clinical isolates over a long period time, thus allowing for a reliable assessment of the resistance trends. In 2010, switching from CLSI to EUCAST guidelines, most antimicrobial susceptibility percentages did not change, although an increase in fluoroquinolones’ susceptibility of *Enterobacter* spp. in the application of the EUCAST criteria was reported, most probably owing to the elimination of the intermediate category [15,16]. *Enterobacter* spp. are intrinsically resistant to ampicillin, amoxicillin, first-generation cephalosporins, and cefoxitin, owing to the presence of a constitutive AmpC β-lactamase. Moreover, the adaptation to the hospital environment, the ability to acquire several genetic mobile elements, and the modulation of the expression of different proteins associated with antimicrobial resistances and virulence make their treatment difficult. In fact, carbapenemases belonging to the New Delhi metallo β-lactamase (NDM) and Verona integron-mediated metallo-β-lactamase (VIM) types have been identified in *E. cloacae* and *K. aerogenes* (*E. aerogenes*) strains in Europe, Asia and America. Serine β-lactamases, such as KPC (*Klebsiella pneumonia* carbapenemases), was also reported, and oxacillinases, such as OXA-48, seem to be the most prevalent in *Enterobacter* spp. Concerning aminoglycosides’ and fluoroquinolones’ resistance, it has been demonstrated that the acquisition of the non-susceptibility phenotype is mediated by transposable elements. Porin defects and augmented levels of efflux pumps are recognized as important elements in multi-drug-resistant strains. Lipopolysaccharide (LPS) modifications are involved in polymyxin susceptibility in *Enterobacter* spp., where alterations in outer membrane structure lead to a considerable decrease in antibiotic activity. In fact, owing to the previous treatments, most *Enterobacter* spp. isolates become resistant to third-generation cephalosporins, penicillins, and fluoroquinolones [6,9].

Over the whole study period, we observed significant increasing and decreasing trends in antibiotic resistance rates of *E. cloacae* and *K. aerogenes* (*E. aerogenes*) clinical isolates among both inpatients and outpatients. Considering the comparison between the periods of 2000–2009 and 2011–2020, the overall resistance rates of *E. cloacae* strains increased for fosfomycin, in both community and hospital-related infections, whereas they decreased for fluoroquinolones and aminoglycosides, principally in the hospitalized population. On the other hand, the overall resistance rates of *K. aerogenes* (*E. aerogenes*) strains increased for Fosfomycin in both groups, increased for aminoglycosides and third-generation cephalosporins in the hospitalized population, and decreased for fluoroquinolones in both outpatients and inpatients. Our data agree with the Italian surveillance report 2015–2021, which described that, among Enterobacterales, particularly *Escherichia coli*, antimicrobial resistance trends decreased for fluoroquinolones and aminoglycosides [17]. Moreover, the European surveillance report of antimicrobial resistance in the same period described that the highest EU/EEA resistance percentages in *Escherichia coli* were observed for aminopenicillins, followed by fluoroquinolones, cephalosporins, and aminoglycosides, although their trends significantly decreased [18]. The differences observed between the hospitalized population and outpatients were most probably due to the different antimicrobial treatments used in hospital compared with those administered in community, as most of the *E. cloacae* and *K. aerogenes* (*E. aerogenes*) strains isolated in the hospital were resistant to more than one class of antibiotic. The antimicrobials most commonly administered in *Enterobacter* infections include fourth-generation cephalosporins, such as cefepime and cefpirome; carbapenems, which seem more effective, even if the use of carbapenems should be rigorously restricted; the piperacillin–tazobactam combination, which presents good treatment results; and aminoglycosides, particularly amikacin, which remain active against more than 95% of *Enterobacter* strains, according to the Infectious Diseases Society of America (IDSA) guidelines [6,9,19,20].

In Italy, fluoroquinolones were the most common antibiotic prescribed in 2019, particularly in the treatment of UTI, preceded only by β-lactams and macrolides [21]. In 2018, the European Medicines Agency (EMA) restricted the usage of fluoroquinolone-containing antibiotics, such as ciprofloxacin, owing to severe, disabling, and potentially permanent side effects, in accordance to following the Pharmacovigilance Risk Assessment Committee (PRAC) recommendations [22]. In 2019, Italy followed these recommendations and our data confirmed the decreasing resistance trends for fluoroquinolones due to a probable diminished clinical usage. As a consequence, the greater administration of aminoglycosides, cephalosporins, and particularly fosfomycin increased the resistance rates to these drugs in *Enterobacter* infections. Fosfomycin, also known as Monuril^®^ or Monurol^®^, is an antibiotic used to treat complicated and uncomplicated UTI and, because most of our samples are infections of the urinary tract, the increasing trend observed was most likely due to an uncontrolled and inappropriate usage. Therefore, our data highlight the importance to follow national and international guidelines in the prudent use of antimicrobials in human health [23]. Moreover, a strong modulation and adequate antimicrobial therapy based on patient’s clinical situation, as well as more attention to the different routes of transmission, which include (I) from environment to patient, (II) from colonized patients to the environment, and (III) between patients, are needed.

Reviewing the literature, our data agreed with the results obtained in other studies, including the increased fosfomycin resistance, as detected in *E cloacae* strains by Jimenez-Guerra and coauthors [12]; the low carbapenem resistance, particularly imipenem, as also described by Jimenez-Guerra and coauthors and Al-Tawfiq and coauthors in *Enterobacter* spp. [10,12]; and high resistance rates to cephalosporins, both third- and four generations, as reported by Quraishi and coauthors [9]. However, Quraishi and coauthors found a percentage of resistance of *Enterobacter* to meropenem of 22%, which disagreed with our results concerning carbapenem non-susceptibility [9]. Carbapenems are the antibiotics used as the first line against strains that produce AmpC beta lactamases, and a high percentage of resistance could be due to possible excessive use in treatments, as previously described [9,10,12]. Moreover, Al-Tawfiq and coauthors observed that nosocomial *E. cloacae* and *E. aerogenes* isolates were statistically more resistant compared with those identified in outpatients, a result similar to our data [10]. As we reported an increased trend of fosfomycin resistance due to inappropriate use in urinary infection, Adhikari and coauthors also described that, in Iran, most urinary *Enterobacter* isolates were resistant to nitrofurantoin, which was incorrectly used to treat these types of infections without an analysis of susceptibility patterns [9].

In this study, 111 bacterial strains were identified as *Enterobacter* spp. using commercialized systems, such as Vitek 1 and 2, based on biochemical tests, and Vitek MALDI-TOF MS, based on mass spectrometry. These techniques present limitations in species’ identification, particularly within the *E. cloacae* complex and between *E. hormaechei*, *E. cloacae*, *E. asburiae*, *E. kobei*, and *E. ludwigii*, owing to the high similarity between them [6]. A precise identification of the species is important for correct antibiotic therapy and misidentifications could have a negative impact on patient outcomes. Molecular biology methods, such as sequencing of 16S rRNA, real-time PCR, and microarray comparative genomic hybridization, are able to identify the species exactly, but these are time-consuming, expensive, and difficult to perform routinely in the laboratory [6].

Our study presents a few limitations that should be considered: (A) the work is retrospective and was performed in a single centre; (B) the lack of clinical data cannot provide a more comprehensive representation of resistance trends; and (C) the unavailability of the previous antimicrobial therapies.

## 4. Materials and Methods

### 4.1. Study Design and Setting

In this retrospective study, antibiotic resistance patterns of *E. cloacae* and *K. aerogenes* (*E. aerogenes*) non-duplicate strains were analyzed. Data were retrieved from the database of the Laboratory of Microbiology of Desio Hospital, Italy, from 1 January 2000 to 31 December 2019. In the case of multiple *E. cloacae* and *K. aerogenes* (*E. aerogenes*) isolates in one subject, showing the same antibiotic resistance pattern, only the first one was used for the analysis. Specimens presenting multiple isolates other than *E. cloacae* and *K. aerogenes* (*E. aerogenes*) were excluded.

### 4.2. Bacterial Isolates and Antimicrobial Susceptibility Testing

Antimicrobial susceptibility patterns of *E. cloacae* and *K. aerogenes* (*E. aerogenes*) non-duplicate isolates were determined by the VITEK^®^ 1 and 2 systems (bioMérieux, Marcy l’Étoile, France) using antimicrobial susceptibility testing (AST) cards. For this retrospective study, resistances to the following 10 antibiotics were analyzed: amikacin, amoxicillin/clavulanic acid, cefotaxime, ceftazidime, ciprofloxacin, norfloxacin, fosfomycin, gentamicin, imipenem, and trimethoprim-sulfamethoxazole. From 2000 to 2010, the results were interpreted using the criteria recommended by the Clinical and Laboratory Standards Institute (CLSI) [24]. From June 2011 to December 2019, the results were interpreted using the criteria recommended by the European Committee on Antimicrobial Susceptibility Testing (EUCAST) [25]. The identification of bacteria was performed by VITEK^®^ 1 and 2 systems and, from 2014, by Vitek^®^ matrix-assisted laser desorption/ionization time-of-flight mass spectrometry (MALDI-TOF MS) (bioMérieux, Marcy l’Étoile, France). *Escherichia coli* ATCC 8739 was used as a control.

### 4.3. Definition

We defined an *E. cloacae* and *K. aerogenes* (*E. aerogenes*) isolate as multi-drug-resistant (MDR) if it exhibited a non-susceptibility to at least one agent in three or more antimicrobial categories. Resistant and intermediate resistant *E. cloacae* and *K. aerogenes* (*E. aerogenes*) isolates were combined, as previously reported [26].

### 4.4. Statistics

All statistical analyses were performed using Stata (Stata Statistical Software: Release 16) [27]. A chi-square test was applied to compare the antimicrobial susceptibilities among inpatient and outpatient results over the study period, which was divided into two intervals of time, 2000–2010 and 2011–2019. The Cochran–Armitage trend test was used to assess for significant trends in antimicrobial susceptibilities, using a two-sided *p*-value with a cutoff of ≤0.05. A *p*-value <0.05 was considered statistically significant.

## 5. Conclusions

Collectively, the major strength of our work is the large sample size and long study period with which we carried out our analyses. Among inpatients and outpatients, we observed differences in the antimicrobial susceptibilities, most probably due to a different treatment or a switch from one antibiotic to another. Therefore, it is important to continuously study and monitor antibiotic non-susceptibilities at local and regional levels, allowing the reduction in inappropriate antibiotic consumption, the detection of new resistance mechanisms, and the introduction of control measures.

## Data Availability

The data presented in this study are available on request from the corresponding author.
